# Effect of Urolithin A on Bovine Sperm Capacitation and In Vitro Fertilization

**DOI:** 10.3390/ani14182726

**Published:** 2024-09-20

**Authors:** Manuela Jorge, Filipa C. Ferreira, Carla C. Marques, Maria C. Batista, Paulo J. Oliveira, F. Lidon, Sofia C. Duarte, José Teixeira, Rosa M. L. N. Pereira

**Affiliations:** 1 Unit of Biotechnology and Genetic Resources, National Institute of Agrarian and Veterinary Research, Quinta da Fonte Boa, 2005-048 Vale de Santarém, Portugal; manuela.pjorge@gmail.com (M.J.); filipa.ferreira@iniav.pt (F.C.F.); carla.marques@iniav.pt (C.C.M.); batista.sao@gmail.com (M.C.B.); 2 Department of Veterinary Sciences Research Centre, Vasco da Gama University School, Lordemão University Campus, 3020-210 Coimbra, Portugal; sofia.duarte@euvg.pt; 3 GeoBioTec—Faculty of Sciences and Technology, New University of Lisbon, 2829-516 Caparica, Portugal; fjl@fct.unl.pt; 4 CNC—Centre for Neurosciences and Cell Biology, University of Coimbra, 3004-504 Coimbra, Portugal; pauloliv@cnc.uc.pt (P.J.O.); jteixeira@cnc.uc.pt (J.T.); 5 CIBB—Center for Innovative Biomedicine and Biotechnology, University of Coimbra, 3004-504 Coimbra, Portugal; 6 LAQV, REQUIMTE, Laboratory of Bromatology and Pharmacognosy, Faculty of Pharmacy, University of Coimbra, Polo III, 3000-548 Coimbra, Portugal; 7 CIISA, Faculty of Veterinary Medicine, University of Lisbon, Av. da Universidade Técnica, 1300-477 Lisboa, Portugal; 8 Associated Laboratory for Animal and Veterinary Science (AL4AnimalS), Av. da Universidade Técnica, 1300-477 Lisboa, Portugal

**Keywords:** urolithin A, spermatozoa, oocyte, ROS, mitochondria, ART, reproductive biotechnologies

## Abstract

**Simple Summary:**

Sperm cells are specialized cells that are highly susceptible to different kinds of damage, impairing the success of in vitro fertilization and, consequently, of assisted reproductive techniques (ART). Oxidative stress plays a significant role in this context causing DNA injury, reduced motility, and compromising the fertilization ability of sperm. The use of antioxidants aims to mitigate the oxidative stress, protect sperm cells from damage, and enhance their fertilization potential. Urolithin A (UA) is a natural compound with proven anti-aging and antioxidant effects in various cells, but its effect on bovine sperm has not been tested yet. The objective of this study was to investigate the impact of UA on bovine sperm function and fertilization rate, thus contributing to animal reproductive development. The results showed that UA improved sperm motility quality and reduced oxidative stress levels in a dose-dependent manner. The use of UA as an antioxidant therapy may have a significant impact on the advancement of reproductive biotechnologies.

**Abstract:**

Reactive oxygen species (ROS) play a critical role in the functional competence of sperm cells. Conversely, excessive generation of ROS can impair sperm function, including their fertilization ability. Urolithin A (UA), a gut bacteria-derived metabolite produced from the transformation of ellagitannins, with anti-aging and antioxidant properties, was investigated for the first time in bovine sperm cells in the present study. Firstly, different doses of UA (0, 1, and 10 μM; 8–16 sessions) were used during the capacitation process of frozen-thawed bovine sperm. Sperm motility was assessed using optical microscopy and CASA. Sperm vitality (eosin-nigrosin), ROS, and ATP levels, as well as mitochondrial membrane potential (JC1) and oxygen consumption were evaluated. A second experiment to test the effect of different doses of UA (0, 1, and 10 μM; 9 sessions) in both the capacitation medium, as above, and the fertilization medium, was also implemented. The embryonic development and quality were evaluated. UA, at a concentration of 1 μM, significantly improved sperm movement quality (*p* < 0.03). There was a trend towards an increase in the oxygen consumption rate (OCR) of capacitated sperm with 1 μM and 10 μM UA supplementation. Moreover, an increase in ATP levels (*p* < 0.01) was observed, accompanied by a reduction in ROS levels at the higher UA concentration. These results suggest that UA may enhance spermatozoa mitochondrial function, modifying their metabolic activity while reducing the oxidative stress. Also, the number of produced embryos appears to be positively affected by UA supplementation, although differences between the bulls may have mitigated this effect. In conclusion, presented results further support previous findings indicating the potential therapeutic value of UA for addressing reproductive sub/infertility problems and improving ART outcomes. In addition, our results also reinforce the important bull effect on ART and that male sperm bioenergetic parameters should be used to predict spermatozoa functionality and developmental potential.

## 1. Introduction

**Figure 1:**
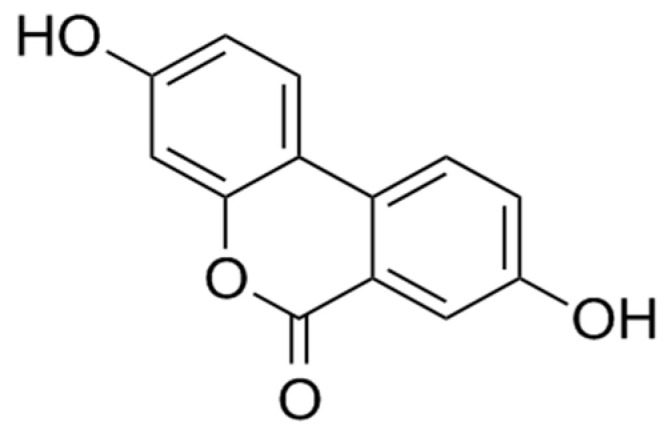
Chemical structure of the urolithin A (UA; modified from https://pubchem.ncbi.nlm.nih.gov/compound/5488186, accessed on 13 September 2024).

Urolithins are natural compounds produced by the gut microbiota from ellagic acid and ellagitannins found in fruits such as pomegranates, strawberries, and nuts [1]. Urolithin A (UA), also known as 3,8-Dihydroxyurolithin (C_13_H_8_O_4_) (Figure 1), is one of the most important compounds in this group [1]. Recently, several tests were conducted to investigate the activity and safety of UA isoforms. In vitro and in vivo studies (mice and human) have shown that UA has a good bioaccessibility and safety profile, with the potential to modulate oxidative stress and recover tissue damage through different mechanisms [2,3,4,5,6,7].

The impact of UA on the prevention of aging (bovine oocytes [8]) and disease processes was postulated, as it enhances mice and human cellular health by increasing mitophagy and mitochondrial function [9]. Mitochondria are organelles that have been described as the “powerhouses of the cells” but are also responsible for producing reactive oxygen species (ROS) [10]. The overproduction of ROS, together with a failure of balance in the body’s antioxidant enzyme systems, can result in a situation of oxidative stress that is a determining factor in the etiology of many pathologies, including the reproductive ones [11,12,13]. Indeed, dysfunctional mitochondria cannot provide the energy needed by mammalian cells, particularly those with high energy consumption, such as spermatozoa [11].

Conversely, the oxidative stress has a critical role in the life and death of specialized mammalian spermatozoa. Spermatozoa mitochondria generate ROS during oxidative phosphorylation, which plays a vital role in the signaling pathways essential for sperm fertilizing ability. Notwithstanding, these cells can also produce ROS from three different sources, which include sperm mitochondria, cytosolic L-amino acid oxidases, and plasma membrane nicotinamide adenine dinucleotide phosphate oxidases (NOXs). Physiological levels of ROS promote sperm capacitation, while excess ROS exposure, or depleted antioxidant defenses, yields a state of oxidative stress, which disrupts their fertilizing capacity [14,15]. ROS-induced spermatozoa changes include the activation of extracellular signal-regulated kinase-like proteins, up-regulation of tyrosine phosphorylation in the tail, and the induction of sterol oxidation, which are triggered by the stimulation of a cyclic adenosine monophosphate (AMPc)/protein kinase A phosphorylation cascade [14]. However, excessive generation of ROS can lead to lipid peroxidation, which disrupts membrane characteristics that are essential for maintaining sperm function, including their ability to fertilize oocytes in mice, bovine, and humans [8,9]. Furthermore, lipid aldehydes produced due to lipid peroxidation can bind to proteins in the mitochondrial electron transport chain, triggering a cycle of further ROS generation. Ultimately, the high levels of oxidative stress can damage the DNA in the spermatozoon nucleus [14]. Guanine is the most readily oxidized of the four DNA bases, being converted to the mutagenic form 8-hydroxy-deoxyguanosine (8-OHdG) [14,15]. Overall, this evidence indicates sperm sensitivity to this type of stress that can affect every aspect of the reproductive process from gametogenesis to the birth of healthy offspring [14,16].

Sperm cells require a balance of oxidants and antioxidants to function properly, and sperm preservation methods can cause oxidative stress (horse) [17]. There are still challenges associated with semen cryopreservation and the harmful effects of oxidative stress on mammalian sperm quality. During cryopreservation, excessive ROS production leads to mitochondrial dysfunction, which causes a decrease in mitochondrial membrane potential and altered sperm functions. The processes of osmotic and thermal stress, as well as intracellular ice crystal formation, also contribute to this excessive ROS production. These events potentiate cryoprotectant-induced calcium overload in the mitochondrial matrix, triggering the opening of mitochondrial permeability transition pores and resulting in the release of pro-apoptotic factors from the mitochondria, initiating an apoptotic cascade [18].

According to Fonseca et al. [8], UA was tested for the first time in animal reproduction, namely in bovine oocytes subjected to in vitro maturation. In this cited study, a model for aging the female gamete was implemented and the negative effect of oocyte aging on its developmental competence was confirmed. The model proved to be very efficient and useful regarding reproductive diseases, gamete ageing, and consequent decreases in fertility. Applying UA supplementation to the maturation medium was established as a new therapeutic approach in assisted reproductive biotechnologies, as UA could prevent or delay female gamete ageing. Moreover, UA has improved blastocyst formation and fertility outcomes. However, UA has never been tested on bovine or other species’ spermatozoa.

The present study intended to evaluate the impact of UA and males on bovine sperm function, fertilization, and embryo production rates. Our approach makes this study innovative, which may contribute to the development of therapies for male sub/infertility and reproductive biotechnologies, an area with growing interest but lacking research.

## 2. Materials and Methods

### 2.1. Experimental Design

**Figure 2:**
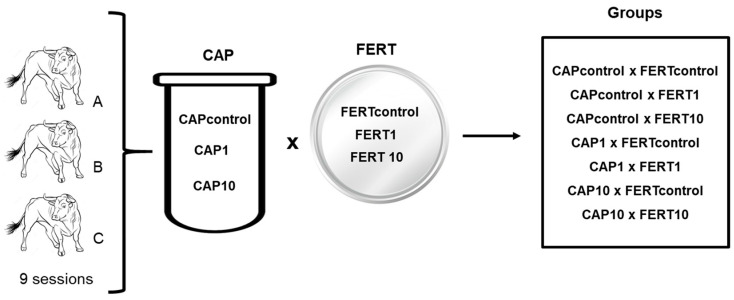
Experimental design of the second experiment to study the effect of UA in the process of the capacitation of bovine spermatozoa and fertilization of oocytes. UA was added to the capacitation medium (CAP) and/or the fertilization medium (FERT) at the concentration of 0 (control), 1, and 10 µM, totalizing seven groups. (A—Bull A; B—Bull B and C—Bull C).

This study was approved by the Ethical Commission of EUVG (nº 15/2022) with the following objectives:

(a) Test the effect of different concentrations of UA (0, 1, and 10 µM; 12–21 replicates) in the capacitation process of frozen-thawed bovine spermatozoa on their morphology, motility, viability, ROS production, and mitochondrial quality. The process of maturation in the capacitation medium (CAP; CAP0—0 µM of UA, CAP1—1 µM of UA, and CAP10—10 µM of UA) was carried out by the swim-up method with spermatozoa from three Holstein-Friesian bulls of proven fertility (pools of four straws from ejaculates from two collection days).
(b) Test the effect of bulls and different doses of UA in both the capacitation medium, as above, and the fertilization medium (Fert0, Fert1, and Fert10), on sperm fertilizing ability and subsequent early embryo development (9 sessions). For this purpose, bovine ovaries (n = 816) were collected in a local abattoir, immediately after slaughter, in 9 sessions, for oocyte collection (n = 2851). After oocyte selection, oocytes were matured for 22 h and transferred to UA supplemented fertilization medium. Then, mature oocytes were inseminated with the capacitated spermatozoa, with UA at the concentration of 0 (control), 1, and 10 µm, totalizing seven groups (Figure 2). Embryo production was evaluated.

### 2.2. Oocyte Preparation

At an abattoir in Santarém, bovine ovaries were collected in thermos bottles with a solution of Dulbecco’s phosphate buffered saline (PBS) at 37 °C and transported within 2–3 h to the laboratory. The PBS was previously supplemented with 0.15% (*w*/*v*) bovine serum albumin (BSA) and 0.05 mg mL^−1^ of kanamycin. At the laboratory, the ovaries were washed with the same solution and the cumulus oocyte complexes (COCs) were obtained by postmortem follicular aspiration, from a heterogeneous pool of antral follicles, between 2 and 8 mm in size [19]. Then, the collected COCs presenting more than three layers of cumulus cells and homogenous ooplasm were selected for maturation in an incubator at 38.8 °C, with a maximum humidity atmosphere and 5% CO_2_, for 22 h. Maturation medium consisted of TCM-199 without HEPES, with 10% fetal bovine serum (FCS), 10 ng mL^−1^ epidermal growth factor (EGF), 0.1 mL sodium pyruvate, and antibiotic (4 μL mL^−1^ gentamicin) [13].

### 2.3. Spermatozoa Preparation and Analysis

Bovine semen from three bulls of proven fertility was thawed (4 straws for each session, 12–21 replicates) at 37 °C for 30 s. Then, it was carefully deposited in equal doses at the bottom of the tubes containing the CAP consisting of modified Tyrode’s medium without calcium, supplemented with 2.4 mM of caffeine [20,21] that was previously prepared and supplemented with different concentrations of urolithin A (0; 1 and 10 μM). Afterwards, spermatozoa were placed in the incubator for 1 h in an atmosphere of 5% CO_2_, at 38.8 °C with maximum humidity [19,21].

The in vitro capacitation process (swim up) occurs during this period when the most active spermatozoa migrate to the surface of the liquid. The supernatant was collected and centrifuged at 500× *g* for 10 min. The supernatant was again aspirated and discarded. The motility and concentration of spermatozoa were immediately evaluated [13,21].

#### 2.3.1. Evaluation of Spermatozoa Movement

After the swim-up process, semen motility was evaluated to assess their quality, through CASA assay with the ISAS-V1 CASA System (Proiser, Valencia, Spain). This system is a reliable tool that provides precise and accurate information on kinematic parameters [22]. The parameters evaluated were total and progressive motility (%); curvilinear velocity (VCL, μm s^−1^); average path velocity (VPA, μm s^−1^); static (%), slow, medium, and rapid motile spermatozoa (n); straight-line velocity (VSL, μ ms^−1^); amplitude of lateral head displacement (ALH, μm); linearity; straightness; wobble VPA/VCL; and beat cross frequency (BCF, Hz).

#### 2.3.2. Evaluation of Spermatozoa Vitality and Morphology

Spermatozoa vitality and morphology were evaluated using the eosin-nigrosine dye, following a procedure adapted from Pereira et al. [23]. To conduct the analysis, equal amounts of capacitated sperm samples and the dye were mixed on a microscope slide. A cover slip was used to smear the mixture, which was then air-dried and directly examined under immersion oil. At a magnification of 1000× (100× immersion and 10× ocular objectives), at least 100 randomized sperm cells were evaluated in each sample. The cells that remained unstained were classified as live, while those that showed any pink or red coloration were classified as dead. Moreover, the presence of any morphological abnormalities in the sperm’s tail, midpiece, and head was also examined.

#### 2.3.3. Evaluation of Mitochondrial Membrane Potential

The mitochondrial membrane potential (MMP) is a measure of mitochondrial activity and was assessed in this study using a fluorescent probe called JC-1 (Invitrogen, Paisley UK). JC-1 can differentiate between fully functional midpiece mitochondria, which exhibit orange/red fluorescence, and cells with lower inner mitochondrial membrane potential, which exhibit green fluorescence. Spermatozoa were subjected to different treatments (CAPcontrol, CAP1, and CAP10) during the swim-up process and then incubated with JC-1 in a solution containing Hanks, HEPES, and BSA for 30 min at 38.8 °C and 5% CO_2_ in humidified air, in the absence of light. After incubation, sperm samples were placed on warmed glass microscope slides, with coverslips, and examined using a fluorescence microscope (Olympus BX51, Olympus Iberia, Barcelona, Spain) with a blue fluorescence filter (BP 470–490, objective UPlanFI 20×/0.50). One hundred randomized spermatozoa in each sample were examined for staining patterns.

#### 2.3.4. Intracellular ATP Levels

To determine intracellular ATP levels, (1 × 10^6^) swimmed-up spermatozoa from different treatments were seeded in a 96-well plate with a white opaque bottom and cultured in 50 µL of medium. The CellTiter-Glo luminescent cell viability assay (Promega, Madison, WI, USA) was used to measure ATP levels as per the manufacturer’s protocol. In brief, 50 µL of CellTiter-Glo reagent (CellTiter-Glo buffer + CellTiter-Glo substrate) was added to the cells and the mixture was shaken on an orbital shaker for 2 min to induce cell lysis. The luminescence signal was then monitored after incubation at 22 °C for 10 min, using a Cytation 3 reader (Biotek Instruments, Winooski, VT, USA).

#### 2.3.5. Evaluation of Hydrogen Peroxide Content

To evaluate the intracellular oxidative stress, cellular H_2_O_2_ was assessed in (1 × 10^6^) spermatozoa by measuring the oxidation of CM-H_2_DCFDA (5-(and-6)-chloromethyl-2′,7′-dichlorodihydrofluorescein diacetate) using an acetyl ester redox indicator (C6827, ThermoFisher Scientific, Waltham, MA, USA). For this purpose, swimmed-up spermatozoa were seeded in 96-well plates after being treated with CAPcontrol, CAP1, and CAP10. The cells were loaded with 5 μM of CM-H2DCFDA in assay buffer, and fluorescence signals were measured with 520 nm excitation and 620 nm emission wavelengths during 30 min at 38.5 °C, to determine the CM-H2DCFDA oxidation rate in a microplate reader (Cytation 3; BioTek Instruments, Winooski, VT, USA).

#### 2.3.6. Analysis of Cellular Oxygen Consumption Rate

To analyze cellular oxygen consumption rate (OCR), the capacitated spermatozoa (CAPcontrol, CAP1, and CAP10 groups) were added to a pre-coated 96-well plate containing 0.5 mg mL^−1^ concanavalin A (ConA) in a modified synthetic oviductal fluid (SOF) medium [16]. The concentration used was 1 × 10^6^ cells per well, with 5 to 8 wells assigned for each group and bull (20 replicates). The OCR were measured at 37 °C using the Seahorse XF Cell Mito Stress Test Kit in the Seahorse XFe96 Extracellular Flux Analyzer (Agilent Scientific Instruments, Santa Clara, CA, USA) following the manufacturer’s instructions [24].

### 2.4. Oocyte Insemination, Embryo Culture and Development

In this study, spermatozoa (2 × 10^6^ spz mL^−1^) were introduced into droplets of fertilization medium (40 μL), each containing 10 mature oocytes. The in vitro fertilization medium was composed of modified Tyrode’s medium enriched with 5.4 USP mL^−1^ of heparin, 10 mM of penicillamine, 20 mM of hypotaurine, and 0.25 mM of epinephrine, as described by Fonseca et al. [8]. The medium was supplemented with urolithin A (0; 1; and 10 μM). The spermatozoa and oocytes were co-incubated for 20 h at 38.8 °C and 5% CO_2_ in humidified air. Then, denuded presumptive zygotes were cultured in 25 μL droplets of SOF supplemented with minimum essential medium (MEM) non-essential amino acid solution, basal medium eagle (BME) amino acids solution, glutathione, and BSA (6 mg mL^−1^). Embryo culture was maintained at 38.8 °C in a humidified atmosphere with 5% CO_2_, 5% O_2_, and 90% N_2_. Cleavage rates were assessed at 48 h post-insemination and embryo development continued for 10 days in SOF + BSA supplemented with 10% fetal calf serum (FCS) (IVF = day 0) under the same conditions [8].

The assessment of embryo development included cleavage rates as a proportion of cleaved embryos and inseminated oocytes. Days 7 and 8 embryo (morula and blastocysts) developmental rates are used as a proportion of morula and blastocysts and cleaved embryos [19]. In addition, on D7/D8, the quality of embryonic development was assessed and classified based on International Embryo Technology Society (IETS) guidelines as good (grade A), fair (grade B), or bad (grade C) [25].

### 2.5. Statistical Analysis

To analyze the data from sperm parameters, the MIXED procedure (PROC MIXED) of the Statistical Analysis Systems Institute (SAS Inst., Inc., Cary, NC, USA) was used. This procedure employed a mixed linear model that considered the treatment (CAP) and bull as fixed effects, allowing the calculation and comparison of means for each treatment. Sessions were considered a random effect. Embryo production data were analyzed with the procedure GLIMMIX (PROC GLIMMIX) using the binary distribution and the logit as link function. The generalized linear mixed model included bull and treatment (CAP × FERT) as fixed effects and session random effect. Comparisons between groups were performed using the PDIFF test. The PROC POWER of SAS was used to confirm if the number of gametes were sufficient.

GraphPad Prism 8.0 software (GraphPad Software, Inc., San Diego, CA, USA) was used for data analysis of intracellular oxidative stress, ATP levels, and oxygen consumption. The results were presented as means ± standard error of the mean (SEM) for the indicated number of experiments. One-way ANOVA analysis, followed by Dunnet’s post-test, was used to compare groups with one independent variable and determine statistical significance. A significance level of *p* ≤ 0.05 was used to determine statistical differences in the values.

## 3. Results

### 3.1. Effect of UA and Bull in Spermatozoa Kinematic Parameters

Results obtained with the supplementation of the capacitation medium with UA are depicted in Table 1.

Urolithin A at a concentration of 1 µM improved (*p* ≤ 0.05) sperm movement quality evaluated through CASA: linearity (*p* = 0.0009), straightness (*p* = 0.001), wobble (*p =* 0.006), and BCF (*p* = 0.009) when compared to the control group.

Urolithin A at a concentration of 10 µM impaired total motility (*p* = 0.02 and *p* = 0.04) when compared to CAPcontrol or CAP1 groups, respectively. Moreover, higher number of static spermatozoa (*p* = 0.02 and *p* = 0.04) were evaluated in CAP10 compared to CAPcontrol and CAP1, respectively. Lower numbers of the medium and rapid spermatozoa (*p* = 0.002 and *p* = 0.01, respectively) were evaluated in CAP10 than in control (Table 1).

Although all the bulls (A, B, and C) had been selected for AI, significant differences (*p* ≤ 0.05) in sperm quality were identified between them (Table 1). The better kinematic features were observed in bull A (total motility, VCL, VSL, ALH, straightness, and BCF).

### 3.2. Effect of UA and Bull on Semen Concentration, Vitality, and Abnormalities

Sperm quality can be determined by measuring three important parameters: motility, morphology, and vitality [26]. After capacitation with UA, the sperm samples were evaluated for concentration, vitality, as well as for the presence of head, intermediate piece, and tail anomalies. The results obtained showed an effect (*p* = 0.0.1 and *p* = 0.049) of UA at the concentration of 10 μM increasing total defects of spermatozoa when compared to the control group and CAP1, respectively (Table 2). No significant differences were observed in the remaining morphological and vitality parameters. However, differences (*p* ≤ 0.05) were identified between bulls after swim up (Table 2). Bulls A and C showed increased vitality (bull A = 52.7%, bull B = 26.2%, and bull C = 45.2%, *p* ≤ 0.003). Bull C had higher (*p* ≤ 0.04) spermatozoa concentration (74.8 × 10^6^ spz mL^−1^) compared to the other bulls (bull A: 40.5 × 10^6^ spz mL^−1^ and bull B: 33.3 × 10^6^ spz mL^−1^).

### 3.3. Effect of UA and Bull on Mitochondrial Membrane Potential

**Figure 3:**
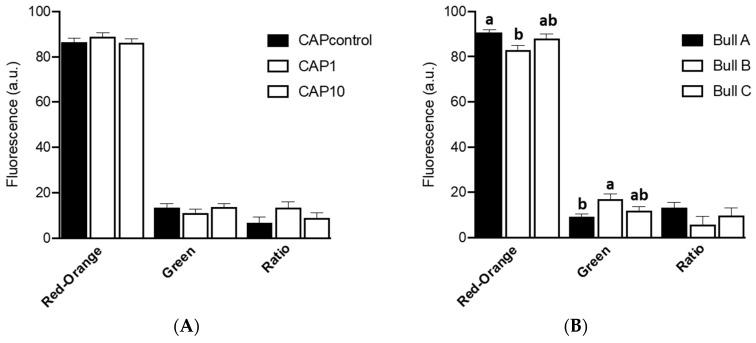
Effect of supplementation of the capacitation medium with UA (**A**) and of bull (**B**) on mitochondrial membrane potential (15 replicates). The data are presented as the mean value± standard error of the mean. CAPcontrol: capacitation medium without supplementation; CAP1: capacitation medium supplemented with 1 μM of UA; CAP10: capacitation medium supplemented with 10 μM of UA. Different letters indicate significant differences (*p* ≤ 0.05).

Analysis of sperm quality can be achieved through accurate measures of sperm mitochondrial functionality [13,16] and are evaluated by measuring the MMP. Results of MMP are represented in Figure 3A without any significant effect of UA (*p* ≥ 0.05). However, bull A has presented more spermatozoa emitting red-orange fluorescence (*p* = 0.01) and few emitting green fluorescence (*p* = 0.004) when compared to bull B (Figure 3B).

### 3.4. Effect of UA and Bull on Spermatozoa Oxygen Consumption Rate, Cellular Oxidative Stress, and Intracellular ATP Levels

**Figure 4:**
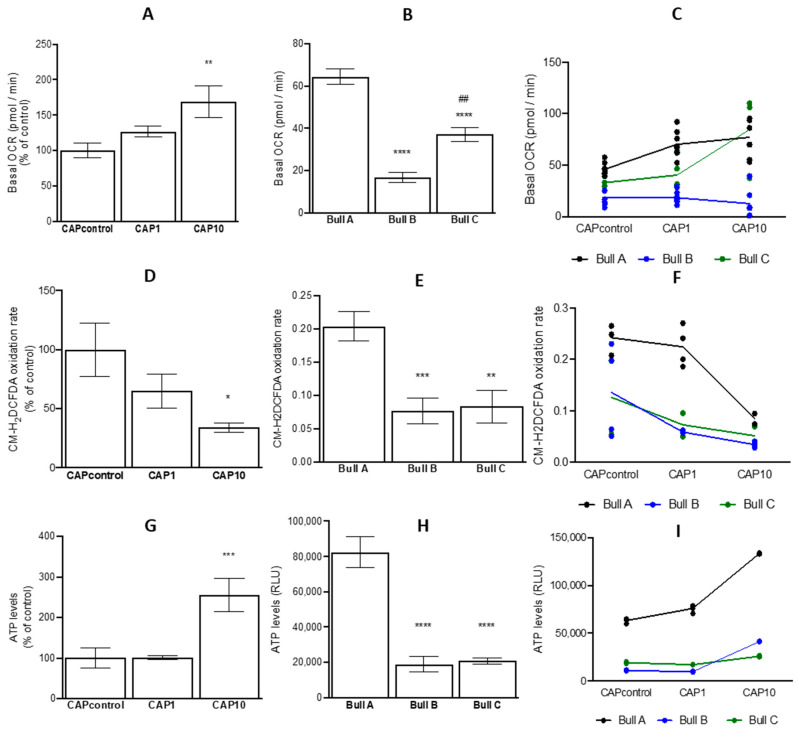
Effect of urolithin A supplementation (0, 1, and 10 μM) and bull, and their interaction, on mitochondrial oxygen consumption rate, ATP levels, and ROS levels during the capacitation process of bovine sperm. (**A**–**C**) Basal oxygen consumption rate (OCR) was analyzed using the Seahorse XFe96 Extracellular Flux Analyzer. Data are means ± standard error of the mean of 21 replicates (5–8 for each bull), and results are expressed in pmol O_2_^−1^ min^−1^ cell mass^−1^. (**D**–**F**) Mean fluorescence signal of the cellular oxidation product CM-H2DCFDA in bovine sperm of different bulls and in the presence or absence of UA supplementation (1 and 10 μM) during the capacitation process. Data are means ± standard error of the mean of 12 replicates (4 each bull), and results are expressed as CM-H2DCFDA fluorescence of sperm (1 × 10^6^). (**G**–**I**) intracellular ATP content in bovine sperm of different bulls and in the presence or absence of UA supplementation (1 and 10 μM) during the capacitation process. Data are means ± standard error of the mean of 12 replicates (4 each bull), and results are expressed as ATP levels per sperm (1 × 10^6^). *, **, *** and **** indicates differences at *p* < 0.1, *p* < 0.05, *p* ≤ 0.01, and *p* < 0.001 respectively, compared to control, or bull A.; CAPcontrol: capacitation medium without supplementation; CAP1: capacitation medium supplemented with 1 μM of UA; CAP10: capacitation medium supplemented with 10 μM of UA. ## indicate significant differences (*p* < 0.05) between Bull B and C.

The OCR is a crucial indicator of mitochondrial activity, which reflects alterations in the electron transport chain [13,27]. Results showed that UA tended to increase basal OCR regardless of the concentration (control = 32.4 ± 4.19 pmol min^−1^; CAP1 = 43.6 ± 7.35 pmol min^−1^; and CAP10 = 53.3 ± 9.45 pmol^−1^ min^−1^) (Figure 4A). A significant effect of the bull (*p* < 0.001) on the OCR of capacitated spermatozoa was identified, with bull A presenting the highest OCR (Figure 4B), that was also the highest for capacitated spermatozoa from control and CPA1 treatments but not for CAP10 (Figure 4C).

Oxidative stress impacts male and female germ lines, with moderate ROS levels benefiting sperm function and the enhancement of sperm capacitation [12]. In the present study, the impact of the UA and bull on oxidative stress in spermatozoa was evaluated after capacitation, using a fluorescent reporter molecule (CM-H2DCFDA). In this case, results showed that UA tended to decrease ROS levels in a dose-dependent manner (control = 100.0 ± 31.02; CAP1 = 81.14 ± 36.80; and CAP10 = 26.12 ± 4.77) (Figure 4D). Bull A has showed the highest ROS level (*p* < 0.05, Figure 4E center) even when considering both effects (bull and UA) (Figure 4F).

Sperm motility hinges on the energy obtained through ATP hydrolysis, which is facilitated by mitochondria located in the sperm’s intermediate piece. Sperm cells acquire their energy from two main pathways: OXPHOS and glycolysis. In the case of bovine sperm, OXPHOS appears to be favored over glycolysis [28,29,30]. After capacitation, UA increased ATP levels in a dose-dependent manner (*p* ≤ 0.01) (Figure 4G), when compared to the control group (control = 100.0 ± 26.6; CAP1 = 104.1 ± 6.9; and CAP10 = 304.4 ± 36.9). In addition, spermatozoa from bull A produced the highest level of ATP (*p* < 0.001, Figure 4H). Also, a different response of spermatozoa to UA per bull was shown (Figure 4I) being the highest levels of ATP identified in bull A.

### 3.5. Effect of UA and Bull on Embryo Production Rates and Quality

**Figure 5:**
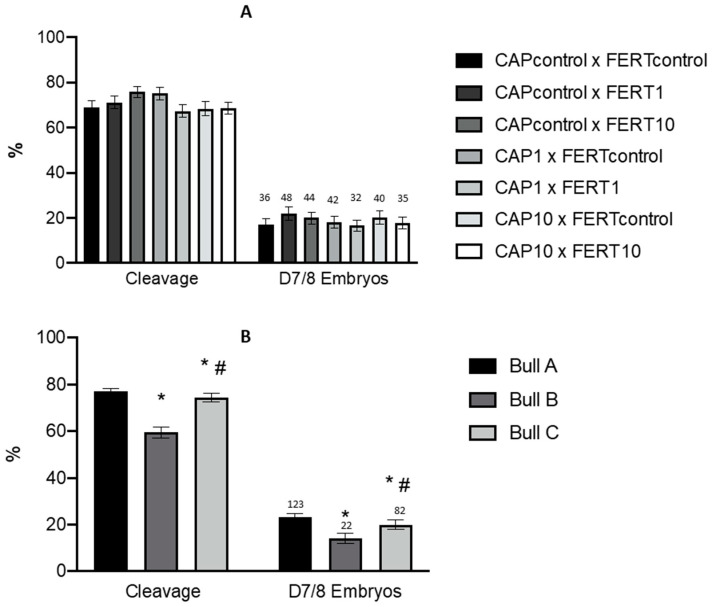
Effect of different concentrations of UA during capacitation and/or fertilization processes (**A**) and bull (**B**) on cleavage and embryo production rates (9 sessions). The data are presented as the mean value ± standard error of the mean and the number (n) of embryos. CAPcontrol: capacitation medium without supplementation; CAP1: capacitation medium supplemented with 1 μM of UA; CAP10: capacitation medium supplemented with 10 μM of UA. FERTcontrol: fertilization medium without supplementation; FERT1: fertilization medium supplemented with 1 μM of UA; FERT10: fertilization medium supplemented with 10 μM of UA. A, B and C represent the three bulls used in this study. * # indicate significant differences (*p* < 0.05). Numbers above bars represent the number of produced embryos.

The results obtained after the supplementation of UA to the CAP medium and/or to the FERT medium are represented in Figure 5. This supplementation did not present a significant influence on the embryo cleavage (*p* ≥ 0.05) and D7/8 embryo production rates (*p* ≥ 0.05) (Figure 5A). In addition, the number of produced embryos (Figure 5, above bars) were 36 in the CAPcontrol × FERTcontrol group and 48 and 44 in the CAPcontrol × FERT1 and CAPcontrol × FERT10 groups, respectively. No significant differences (*p* ≥ 0.05) were observed in the quality rates of the produced embryos.

A significant effect of semen donors on embryo cleavage (*p* < 0.0001) and D7/8 embryos (*p* = 0.0114) rates were clearly identified. The bull B has presented the worst results (*p* < 0.0001 and *p* = 0.01). In addition, bull A showed more embryos of higher quality (grade A) than bull C (*p* = 0.004). Bull C had more embryos of medium quality (grade B) than bull A (*p* = 0.0006). No differences were identified for low quality (grade C) embryos among bulls.

## 4. Discussion

The impact of UA on bovine spermatozoa capacitation and fertilization processes, and embryonic development was demonstrated for the first time in the present study. This antioxidant, at a concentration of 1 μM improved sperm linearity, straightness, wobble, and BCF, and therefore the sperm movement quality. UA was also able to modify the metabolic activity of spermatozoa and reduce their oxidative stress. Moreover, the number of embryos produced appears to be positively affected by UA supplementation, although differences between bulls may have mitigated this effect. In fact, despite that the technology for in vitro embryo production still requires considerable refinement, the production of a higher number of good quality embryos can allow higher gestation rates and therefore, a higher number of neonates [31,32]. These results are of primordial importance for ART success [33].

Despite more than 60 years of optimization of semen cryopreservation protocols, the freezing process remains highly damaging for spermatozoa. In bovine artificial insemination centers, 40–50% of sperm cells do not support cryopreservation, which generates high economic losses [32,34]. Moreover, differences in the male reproductive ability for field reproduction compared to artificial insemination or embryo production, as in the present study, were often reported [23,35]. According to Losano et al., the ability of traditional methods to identify and discriminate semen samples of superior quality is limited, suggesting the characterization of their bioenergetics and kinematic parameters to predict sperm functionality [36]. Although all bulls used in the present study belong to an artificial insemination center, our results clearly showed that higher embryo production rates were obtained with semen from bull A compared to the other males. Accordingly, bull A spermatozoa presented the best MMP, OCR, and ATP results that were reflected in the highest embryo production rates. On the other hand, it is very desirable to have a positive effect of UA supplementation on gametes collected from different animals, especially of those with greater limitations in terms of fertility but also potentiating the best males, as was the case in this study.

Several studies have already shown that UA regulates mitochondrial function, reduces ROS, prevents cellular damage, and various pathologies [2,3,4,6,37]. In the processes of in vitro fertilization, sperm cells, which are specialized cells with high energy consumption, are subjected to excessive ROS production during semen collection, cryopreservation, and laboratory procedures [14,15,18,38,39]. Oxidative stress is not only relevant in sperm but also in oocytes and is a major contributing factor to low oocyte maturation efficiency and deterioration of their quality, resulting in decreased fertilization and embryo production [12]. Therefore, UA, by improving mitochondrial health and respiration, might contribute to improve ART results. However, the biological effects of UA on gametes and embryos remain poorly characterized demanding further studies.

In the context of in vitro fertilization (IVF) systems, various parameters are commonly assessed to evaluate sperm quality, such as morphology, concentration, and motility [26]. However, more refined methodologies can be used to improve this evaluation, such as ATP and ROS production, OCR, and MMP. The primary function of a mitochondrion is to carry out oxidative phosphorylation (OXPHOS), which results in the production of ATP, a vital metabolic energy source required for flagellar beating and sperm motility [40]. In the present study, UA supplementation at the highest concentration induced an increase in ATP production during the capacitation process. However, this dose (CAP10) has also reduced total motility with a decrease in rapid spermatozoa and increase in the static ones. According to Aitken, in 2017 [14], the loss of motility was one of the first and most powerful impacts of oxidative stress on sperm. Conversely, a reduction in ROS levels was identified when spermatozoa were supplemented with the highest UA dose during capacitation. Recently, Santos et al. [13] have shown that the mitochondrial-targeted antioxidant, AntiOxBEN2, supplemented to the capacitation and fertilization media positively influenced both bovine sperm capacitation and fertilization processes, inducing a substantial reduction in ROS production, thereby improving early embryonic development. In accordance, Tiwari et al. have improved the quality of thawed semen by using the mitochondrial-targeted antioxidant called Mito-TEMPOL during cryopreservation [18]. Tripathi et al. [41], also showed that the antioxidant supplementation (melatonin, ascorbic acid, α-tocopherol, sodium selenite) reduced oxidative stress by decreasing ROS levels, thus improving embryo quantity and quality.

According to the results obtained, there was a tendency towards an increase in the OCR of capacitated sperm with 1 μM and 10 μM UA supplementation. As referred, OCR is a crucial indicator of mitochondrial activity reflecting alterations in the electron transport chain. It serves as a representative measure of OXPHOS, the process by which spermatozoa utilized oxygen. In the case of bovine sperm, OXPHOS predominantly serves as their main energy-generating pathway [29,30]. Accordingly, it was shown that an increase in ATP levels of capacitated spermatozoa corresponded with a reduction in ROS levels at the highest concentration of UA. Thus, UA might have increased the mitochondrial function presenting some nuances in a dose-dependent manner. This result is relevant because excessive ROS can lead to oxidative stress and negatively affect sperm function during fertilization [11,14,39]. Interestingly, bull A had the highest ROS level but also the highest MMP, ATP production, and OCR, probably indicating a healthy mitochondrial functionality that was related with higher cleavage and D7-8 embryo production rates. ROS can be originated from both endogenous sources, such as sperm metabolism, and exogenous sources, like ART. Therefore, it is important to maintain an appropriate balance of ROS to support sperm health and functionality [11,14,39,42] that may be varied among male spermatozoa donors and should be further investigated.

Presented results demonstrate, for the first time, the beneficial effect of UA on bovine sperm capacitation and in vitro fertilization. Although no significant differences were found in embryo quality rates, supplementation of CAP or FERT with UA increased the number of produced embryos compared to the control group. The positive effect of UA on the quality of capacitated spermatozoa is noteworthy. These results were obtained using the semen of three bulls with distinct characteristics and demonstrates that UA supplementation benefits gamete development potential, as was already shown by Fonseca et al. [8] in bovine oocyte maturation. It would be interesting to test an intermediate concentration of UA, as the concentration of 10 μM showed better outcomes in reducing ROS and increasing ATP levels, while the concentration of 1 μM has induced better morphological and kinematic parameters, particularly in sperm movement quality.

The importance of ART and the expansion of reproductive biotechnologies in the context of animal production and reproductive efficiency has a significant socio-economic impact worldwide. The selection of males based on fertility is highly significant, as they can contribute valuable genetic material to their offspring, allowing for a greater selection differential compared to females [43]. Moreover, the use of cryopreserved semen contributes to maintain the genetic diversity within a population while mitigating the effects of inbreeding [44]. Therefore, the prediction of male reproductive outcomes allied to extensive implementation of antioxidant therapies, employed for the prevention, treatment, and enhancement of reproductive efficiency, holds unparalleled significance.

## 5. Conclusions

Obtained results showed that supplementation of UA at a concentration of 1 μM improved sperm movement quality, while the highest concentration decreased total motility and spermatozoa speed. A positive trend in increasing the oxygen consumption rate of capacitated sperm with both 1 μM and 10 μM UA supplementation was also identified. Additionally, 10 μM UA supplementation increased ATP and reduced ROS levels in capacitated sperm, indicating an enhanced mitochondrial function. Clearly, UA was able to modify the metabolic activity of spermatozoa and thus reduce their oxidative stress. Although the number of produced embryos may be positively affected by UA, the differences between the bulls may have mitigated this effect. Also, differences in the spermatozoa bioenergetics parameters among the bulls were reflected in embryo developmental rates. These results further support previous findings indicating the potential therapeutic value of UA in addressing reproductive sub/infertility problems and improving ART results, making it a highly relevant and timely topic of interest that demands further research. In addition, the sperm bioenergetic parameters should be used to predict their functionality and developmental potential, and for the selection of bull breeders.

## Figures and Tables

**Table 1:** Effect of bull and supplementation of the capacitation medium with urolithin A (UA) on the kinematic parameters of semen using computer-assisted sperm analysis (CASA, 12 replicates).

Kinematic Parameters	Groups					
	UA Effect			Bull Effect		
	CAPcontrol	CAP1	CAP10	Bull A	Bull B	Bull C
Total Motility (%)	61.2 ± 3.33 ^a^	60.1 ± 3.24 ^a^	55.3 ± 3.33 ^b^	71.7 ± 3.64 ^a^	58.2 ± 5.31 ^b^	46.7 ± 6.23 ^b^
Progressive motility (%)	30.9 ± 4.39	32.1 ± 4.19	32.8 ± 4.37	39.4 ± 4.30 ^a^	35.3 ± 6.49 ^ab^	21.1 ± 7.24 ^b^
VCL (µms^−1^)	67.0 ± 5.67	67.2 ± 5.43	75.8 ± 5.65	83.1 ± 5.64 ^a^	58.1 ± 9.53 ^b^	68.8 ± 8.47 ^ab^
VAP (µms^−1^)	38.3 ± 3.66	41.4 ± 3.58	43.1 ± 3.65	50.3 ± 4.04	37.7 ± 6.92	37.8 ± 5.87
Static spz (n)	37.9 ± 3.34 ^b^	39.0 ± 3.25 ^b^	44.0 ± 3.34 ^a^	27.5 ± 3.66 ^a^	52.6 ± 6.26 ^b^	40.8 ± 5.33 ^b^
Slow motile spz (n)	4.6 ± 0.55	4.7 ± 0.52	3.8 ± 0.54	2.6 ± 0.54 ^b^	1.9 ± 0.91 ^b^	8.5 ± 0.81 ^a^
Medium motile spz (n)	8.8 ± 1.16 ^a^	7.3 ± 1.11 ^ab^	5.3 ± 1.16 ^b^	5.4 ± 1.17 ^b^	2.5 ± 1.97 ^b^	13.4 ± 1.75 ^a^
Rapid motile spz (n)	15.2 ± 1.94 ^a^	12.3 ± 1.87 ^ab^	10.9 ± 1.94 ^b^	12.7 ± 2.03 ^b^	4.9 ± 3.46 ^b^	20.9 ± 3.00 ^a^
VSL (µms^−1^)	32.3 ± 3.61	37.2 ± 3.51	37.1 ± 3.60	44.9 ± 3.86 ^a^	33.3 ± 6.59 ^ab^	28.3 ± 5.69 ^b^
ALH (µm)	2.5 ± 0.15	2.2 ± 0.15	2.3 ± 0.15	2.8 ± 0.15 ^a^	1.8 ± 0.24 ^b^	2.5 ± 0.22 ^a^
Linearity	47.3 ± 1.96 ^b^	55.0 ± 1.83 ^a^	54.0 ± 1.95 ^a^	53.6 ± 1.66	54.5 ± 2.7	48.3 ± 2.64
Straightness	79.0 ± 1.91 ^b^	86.6 ± 1.76 ^a^	83.5 ± 1.89 ^ab^	87.0 ± 1.51 ^a^	81.3 ± 2.44 ^b^	80.8 ± 2.48 ^b^
Wobble VAP/VCL	57.2 ± 1.67 ^b^	62.2 ± 1.58 ^a^	61.9 ± 1.66 ^a^	60.0 ± 1.52	62.0 ± 2.53	59.2 ± 2.35
BCF (Hz)	6.0 ± 0.31 ^b^	7.0 ± 0.29 ^a^	6.6 ± 0.31 ^ab^	7.4 ± 0.25 ^a^	6.3 ± 0.41 ^b^	6.0 ± 0.41 ^b^

The data are represented as estimated mean ± standard error of the mean. Different letters indicate significant differences (*p* ≤ 0.05). VCL: curvilinear velocity; VAP: average path velocity; spz: spermatozoa; VSL: straight-line velocity; ALH: lateral head displacement amplitude; BCF: beat cross frequency. CAPcontrol: capacitation medium without supplement (control); CAP1: capacitation medium supplemented with 1 μM UA; CAP10: capacitation medium supplemented with 10 μM UA.

**Table 2:** Effect of bull and supplementation of the capacitation medium with UA on sperm concentration, vitality, morphology (12 replicates).

Parameters	Groups					
	UA Effect			Bull Effect		
	CAPcontrol	CAP1	CAP10	Bull A	Bull B	Bull C
Concentration (×10^6^ spz mL^−1^)	51.9 ± 7.59	50.4 ± 7.59	46.3 ± 7.59	40.5 ± 7.03 ^b^	33.3 ± 14.92 ^b^	74.8 ± 12.18 ^a^
Vitality (%)	39.7 ± 2.52	44.7 ± 2.49	39.6 ± 2.40	52.7 ± 2.47 ^a^	26.2 ± 4.16 ^b^	45.2 ± 3.48 ^a^
Head defect (%)	7.6 ± 1.12	7.5 ± 1.10	9.7 ± 1.06	7.1 ± 1.03	10.7 ± 1.73	7.2 ± 1.46
Intermediate piece defect (%)	3.0 ± 0.59	4.2 ± 0.58	4.4 ± 0.54	3.1 ± 0.46	5.3 ± 0.74	3.1 ± 0.64
Tail defect (%)	3.8 ± 1.11	4.3 ± 1.09	4.9 ± 1.04	2.4 ± 1.02	3.7 ± 1.70	7.0 ± 1.43
Total anomalies (%)	14.4 ± 1.80 ^b^	15.7 ± 1.78 ^b^	19.0 ± 1.72 ^a^	12.5 ± 1.84	19.7 ± 3.13	17.0 ± 2.60

The data are presented as the mean value ± standard error of the mean. CAPcontrol: capacitation medium without supplementation; CAP1: capacitation medium supplemented with 1 μM of UA; CAP10: capacitation medium supplemented with 10 μM of UA. Different letters indicate significant differences (*p* ≤ 0.05).

## Data Availability

The data presented in this study are available on request to the corresponding author.
